# Liraglutide, a GLP-1 Receptor Agonist, Mitigates LPS-Induced Osteoclastogenesis and Bone Loss by Downregulating Macrophage TNF-α Expression

**DOI:** 10.3390/ijms27125624

**Published:** 2026-06-22

**Authors:** Kou Murakami, Hideki Kitaura, Fumitoshi Ohori, Aseel Marahleh, Angyi Lin, Ziqiu Fan, Kohei Narita, Tomoko Ishiyama, Jin Hu, Huidan Zheng, Hiroyasu Kanetaka

**Affiliations:** 1 Division of Orthodontics and Dentofacial Orthopedics, Tohoku University Graduate School of Dentistry, 4-1 Seiryo-Machi, Aoba-ku, Sendai 980-8575, Japan; kou.murakami.b2@tohoku.ac.jp (K.M.); fumitoshi.ohori.b4@tohoku.ac.jp (F.O.); lin.angyi.d7@tohoku.ac.jp (A.L.); fan.ziqiu.a6@tohoku.ac.jp (Z.F.); kohei.narita.a2@tohoku.ac.jp (K.N.); tomoko.ishiyama.b5@tohoku.ac.jp (T.I.); hu.jin.q5@dc.tohoku.ac.jp (J.H.); zheng.huidan.p4@dc.tohoku.ac.jp (H.Z.); hiroyasu.kanetaka.e6@tohoku.ac.jp (H.K.); 2 Creative Interdisciplinary Research Division, Frontier Research Institute for Interdisciplinary Sciences, Tohoku University, 6-3 Aramaki aza Aoba Aoba-ku, Sendai 980-8578, Japan; aseel.mahmoud.suleiman.marahleh.e6@tohoku.ac.jp; 3 Division of Advanced Dental Science and Technology, Graduate School of Biomedical Engineering, Tohoku University, 6-6-12, Aramaki Aza Aoba Aoba-ku, Sendai 980-8579, Japan; 4 Division of Interdisciplinary Co-Creation (ICC-Division), Liaison Center for Innovative Dentistry, Tohoku University Graduate School of Dentistry, 4-1 Seiryo-Machi, Aoba-ku, Sendai 980-8575, Japan

**Keywords:** bone, osteoclast, liraglutide, bone resorption, diabetes

## Abstract

Liraglutide, a glucagon-like peptide-1 (GLP-1) receptor agonist, restores hyperglycemic conditions in patients with type 2 diabetes and has recently shown promising anti-inflammatory properties. In this study, we explored its potential to suppress osteoclast formation and bone loss triggered by lipopolysaccharide (LPS), an inflammatory agent. In animal models, the co-administration of liraglutide with LPS on the calvaria regions in mice markedly reduced osteoclast numbers and bone resorption areas relative to treatment with LPS alone. Furthermore, the expression levels of receptor activators of the NF-κB ligand (RANKL) and tumor necrosis factor (TNF)-α mRNA were notably lower in the group receiving liraglutide and LPS compared to treatment with LPS alone. Moreover, in vitro tests revealed that liraglutide has no direct inhibitory effect on RANKL-induced osteoclastogenesis and TNF-α-induced osteoclastogenesis. In addition, liraglutide had no direct inhibitory effect on LPS-stimulated RANKL expression in osteoblasts. Moreover, liraglutide effectively suppressed TNF-α mRNA expression in macrophages stimulated by LPS. These findings suggest that liraglutide prevents inflammatory bone destruction not by targeting osteoclast formation directly but by inhibiting the production of TNF-α within macrophages.

## 1. Introduction

Recently, the incidence of chronic metabolic conditions has surged globally; this alarming increase is primarily attributed to both modern lifestyle habits and environmental factors. In addition, there has been a profound escalation in the rates of clinical obesity, impaired blood sugar regulation, persistently high blood pressure, and abnormal cholesterol profiles worldwide. Emerging clinical evidence has highlighted a critical link between metabolic health and bone integrity [1,2,3]. The worldwide escalation of type 2 diabetes cases has solidified its status as a critical public health crisis in the 21st century [4,5,6]. This clinical observation is often termed the diabetic bone paradox because this increased risk persists even when the bone mineral density (BMD) is normal or paradoxically high [7]; the underlying mechanisms involve the deterioration of bone quality. This risk may be further controlled by the pharmacological interventions used to manage glycemia. Specifically, thiazolidinediones (TZDs) have been strongly linked to reduced BMD and increased fracture risk because they promote mesenchymal stem cell differentiation into osteoblasts rather than adipocytes [8,9]. Similarly, human recombinant insulin therapy has been associated with higher fracture rates, potentially due to an increased frequency of hypoglycemia-related falls [10]. Conversely, metformin has demonstrated a protective effect, with several studies suggesting that it may enhance osteoblast differentiation and contribute to a decreased fracture risk in diabetic populations [11,12].

The recruitment of osteoclasts is a critical driver of systemic and localized bone erosion in diseases such as rheumatoid arthritis (RA) [13,14]. This process involves receptor activator of NF-κB ligand (RANKL) and macrophage colony-stimulating factor (M-CSF)—both of which are essential for the survival and maturation of osteoclast precursors [15]. In addition to this pathway, tumor necrosis factor (TNF)-α has been identified as a significant independent inducer of osteoclast formation in vitro [16,17]. TNF-α acts synergistically with RANKL to promote the expression of osteoclast-specific genes, such as tartrate-resistant acid phosphatase (TRAP) and cathepsin K, which facilitate the degradation of the mineralized bone matrix [18,19]. TNF-α not only promotes the differentiation of precursors but also significantly enhances their sensitivity to low levels of RANKL [20,21]. Increases in TNF-α levels lead to osteoclast formation and bone destruction in pathological conditions such as RA and periodontitis. Furthermore, mechanical stress such as orthodontic tooth movement also induce elevated TNF-α levels and therefore promote osteoclast formation and bone resorption [22,23].

Lipopolysaccharide (LPS) is a powerful activator of the toll-like receptor 4 complex. Upon binding to this receptor, LPS triggers an intracellular signaling cascade that activates the innate immune system through a surge in the systemic and localized production of proinflammatory mediators, most notably TNF-α, secreted by macrophages and various other leukocyte populations [24]. The chronic elevation of these cytokines plays a critical role in the development and progression of inflammatory osteoporosis [25]. In such pathological states, the inflammatory milieu disrupts the bone remodeling process. Specifically, the overabundance of TNF-α and related factors shifts the equilibrium toward excessive bone resorption by stimulating osteoclast activity while simultaneously inhibiting the bone-forming capabilities of osteoblasts. Consequently, the immune response initiated by LPS serves as a molecular bridge between bacterial infection and the degradation of skeletal integrity. RANKL and TNF-α have been identified as central mediators of osteoclast differentiation triggered by LPS and the resulting bone destruction [26,27,28]. Moreover, LPS directly influences osteoblasts, thereby triggering a signaling response that significantly enhances the synthesis and subsequent release of RANKL [29]. RANKL promotes osteoclast formation and maturation by binding to RANK on osteoclast precursor cells and serves as a primary mediator of bone resorption [30]. Therefore, LPS-induced inflammation is thought to promote osteoclastogenesis and bone resorption via two distinct pathways: one mediated by inflammatory cytokines such as TNF-α and another mediated by RANKL [29,31].

Glucagon-like peptide-1 (GLP-1), an incretin hormone, is a fundamental regulator of glucose homeostasis. GLP-1 has a known immediate impact on stabilizing blood sugar concentrations and enhances the population and functional capacity of pancreatic islets, which are responsible for insulin synthesis and secretion [32,33]. Mice deficient in GLP-1 receptors elevate osteoclast formation and show osteopenia, indicating that GLP-1 signaling naturally suppresses bone resorption [34]. GLP-1 actively promotes bone formation and contributes to a positive metabolic balance in skeletal tissues. Enhanced bone formation triggered by GLP-1 receptor activation has previously been documented in both insulin-resistant, fructose-fed and streptozotocin-induced diabetic rat models [35].

Increased bone resorption triggered by antidiabetic therapies such as TZDs may lead to a higher likelihood of fractures [8,36,37]. In contrast, GLP-1 receptor agonists have been shown to stimulate osteoblast activity and suppress osteoclast-mediated bone breakdown, and this dual mechanism of action makes the GLP-1 pathway a highly effective candidate for drug development. This suggests that developing GLP-1 signaling modulation approaches may revolutionize the treatment landscape for osteoporosis and other destructive osteolytic disorders. As the first approved GLP-1 receptor agonist for diabetes management, the reported clinical background of exendin-4 lends additional weight to this hypothesis [38]. Despite possessing structural and functional features similar to those of native GLP-1, exendin-4 resists degradation by dipeptidyl peptidase-4 (DPP-4), an enzyme that swiftly cleaves natural GLP-1 in systemic circulation [39]. Due to its significantly extended biological half-life, optimized pharmacokinetic profile, and remarkable therapeutic potency, exendin-4 is an exceptionally viable candidate for diverse clinical applications, offering a more sustainable and effective treatment modality than its predecessors [39,40,41]. We have previously established that exendin-4 mitigates LPS-induced bone loss and osteoclast differentiation in animal models [42], as well as osteoclastogenesis during orthodontic tooth movement [43]. Similarly, linagliptin, a DPP-4 inhibitor, suppresses LPS-induced osteoclastogenesis and bone resorption in vivo [44] and inhibits osteoclastogenesis during orthodontic tooth movement [45].

As a GLP-1 receptor agonist, liraglutide is 97% identical to human GLP-1. Whereas the native peptide has a short half-life, liraglutide features a single amino acid substitution of lysine with arginine at position 34 (Arg^34^) and the attachment of a C-16 fatty acid chain (palmitate) via a γ-glutamic acid linker to lysine 26 (Lys^26^). This molecular design enables its reversible binding to serum albumin and the formation of a self-assembled heptameric structure, which collectively delay subcutaneous absorption and protect the peptide from rapid DPP-4 degradation [46]. Moreover, liraglutide exhibits a low immunogenicity risk. The activation of these receptors promotes insulin secretion in response to glucose and lowers glucagon levels to help manage blood sugar levels [47]. Additionally, it delays gastric emptying, enhances the feeling of satiety, and reduces overall food intake, thus supporting weight loss [48,49]. Moreover, liraglutide affects bone metabolism, potentially by promoting bone formation [50]. Clinical studies have suggested that liraglutide can promote the expression of the bone formation marker P1NP and help maintain BMD [50]. While liraglutide alone may slightly reduce BMD due to weight loss, combining it with exercise has been shown to be effective for preserving bone health in areas such as the hip and spine [50,51]. Recent pharmacological studies have highlighted the potential bone-protective effects of liraglutide. Specifically, the evidence suggests that liraglutide directly suppresses the differentiation of bone marrow macrophages and RAW264.7 cells into osteoclasts. This inhibitory effect is showed to be mediated through the downregulation of key signaling pathways, namely the nuclear factor kappa-B (NF-κB) and mitogen-activated protein kinases (MAPKs), both of which are essential for osteoclastogenesis [52]. Although it has been established that GLP-1 receptor mRNA is highly expressed in tissues such as the lungs, heart, and pancreatic islets, GLP-1 receptor expression was not identified in murine osteoblasts or osteoclasts [53]. This discrepancy raises a critical question: If the receptor is absent in the bone tissue, how does liraglutide exert its effects? Consequently, it remains unclear whether liraglutide inhibits osteoclast formation through direct, receptor-mediated action on bone cells or via an indirect systemic pathway. Further investigation is required to reconcile these findings and clarify the therapeutic potential of GLP-1 analogs for the management of bone-resorptive diseases.

These observations imply that liraglutide modulates bone turnover by regulating osteoclast development and subsequent bone loss under inflammatory conditions. Therefore, we established a murine model to evaluate the effect of liraglutide intervention on LPS-triggered bone resorption and osteoclast differentiation.

## 2. Results

### 2.1. In Vivo Suppression of LPS-Induced Osteoclast Differentiation by Liraglutide

**Figure 1:**
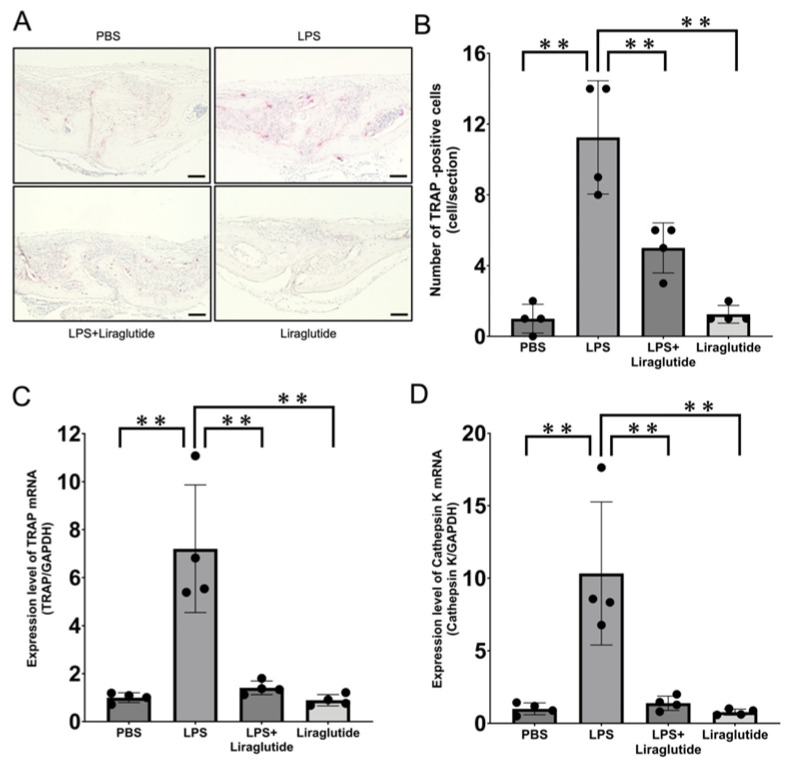
Liraglutide diminished LPS-triggered osteoclast formation in a murine model. (**A**) Micrographs showing TRAP staining of mouse calvarial tissue sections after a 5-day subcutaneous treatment regimen with PBS, LPS, LPS plus liraglutide, or liraglutide independently. Hematoxylin was utilized for counterstaining. Scale bar = 50 μm. (**B**) Quantitative analysis of multinucleated TRAP-positive cells within the mesenchymal area of the sagittal suture. qPCR analysis showing the relative transcript levels of (**C**) TRAP and (**D**) Cathepsin K in calvarial bone fragments, with normalization to GAPDH. Statistics: one-way ANOVA followed by the Tukey–Kramer post hoc test; results are mean ± standard deviation (SD; n = 4; ∗∗ *p* < 0.01).

To evaluate the potential of liraglutide to modulate LPS-triggered osteoclast formation in vivo, we locally injected LPS in the presence and absence of liraglutide into the supracalvarial regions of model mice. Histopathological examination conducted after five days of consecutive treatment demonstrated that LPS administration induced a robust osteoclastogenic response. This was evidenced by the dense accumulation of large multinucleated TRAP-positive cells localized primarily within the mesenchymal tissues of the cranial sutures. A comparative assessment showed that the LPS-induced increase in osteoclast numbers triggered by LPS was effectively countered in the liraglutide co-administration group. Specifically, the liraglutide-treated group exhibited a significantly lower mean number of osteoclasts than that in the group treated with LPS alone, with the numbers of TRAP-positive multinucleated cell decreasing from 11.25 ± 3.20 cells/section in the LPS group to 5.50 ± 1.91 cells/section in the LPS + liraglutide group. In contrast, the phosphate-buffered saline (PBS) and liraglutide-alone groups showed low osteoclast numbers of 1.00 ± 0.82 and 1.25 ± 0.50 cells/section, respectively (Figure 1A,B). To further validate the biological evidence for osteoclastogenesis, we evaluated the expression profiles of key osteoclastogenic genes. Although the LPS-challenged group demonstrated a sharp elevation in the mRNA transcripts of TRAP and cathepsin K, these pathological increases were notably diminished in subjects receiving liraglutide. TRAP mRNA expression increased from 1.00 ± 0.20 in the PBS group to 7.20 ± 2.66 in the LPS group and was reduced to 1.41 ± 0.28 in the LPS + liraglutide group. Similarly, cathepsin K mRNA expression increased from 1.00 ± 0.41 to 10.33 ± 4.93 following LPS treatment and was reduced to 1.39 ± 0.49 by liraglutide co-administration (Figure 1C,D). These results indicated that liraglutide exerts a protective effect by downregulating the genetic signaling pathways essential for osteoclast formation.

### 2.2. In Vivo Suppression of LPS-Induced Bone Resorption by Liraglutide

**Figure 2:**
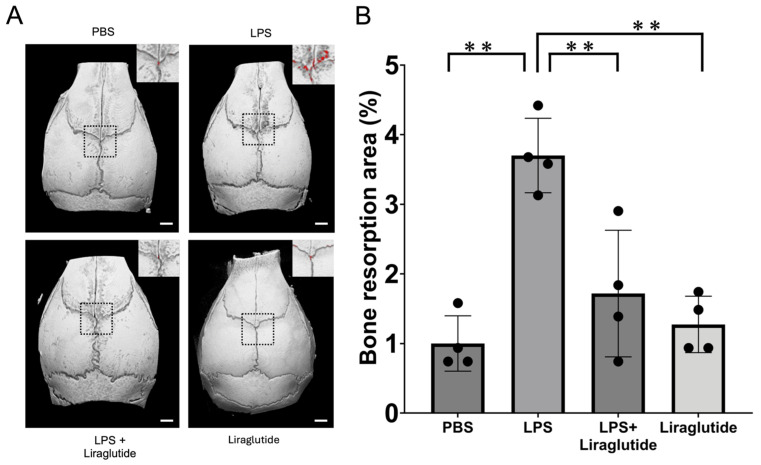
Liraglutide attenuated LPS-mediated bone loss in vivo. (**A**) Representative micro-CT 3D reconstructions of murine calvariae following 5-day subcutaneous administration of PBS, LPS, LPS combined with liraglutide, or liraglutide alone. Areas of bone resorption are highlighted in red. Scale bar = 1.0 mm. (**B**) Morphometric quantification of bone destruction, represented as the ratio of resorbed surface area to a region of interest. Statistics: one-way ANOVA followed by the Tukey–Kramer post hoc test; results are mean ± SD (n = 4; ∗∗ *p* < 0.01).

The structural integrity of the mouse calvariae was rigorously assessed through μCT imaging for its high-resolution capacity to detect surface erosion. Our findings indicate that the LPS challenge triggers an aggressive osteoclastic response, resulting in extensive and visible osteolytic lesions throughout the calvarial bone. The quantitative data mirrored these morphological observations; the ratio of resorbed bone area to total surface area was substantially greater in LPS-challenged subjects (3.70 ± 0.53%) than in either the PBS (1.00 ± 0.40%) or liraglutide baseline groups (1.27 ± 0.40%). Bone loss was dramatically attenuated in the group that concurrently received LPS and liraglutide, with the resorbed bone area reduced to 1.72 ± 0.91%. This reduction in the resorbed area suggests that liraglutide effectively counteracted the catabolic effects of LPS, significantly preserving cranial bone resorption compared to the group treated with LPS alone (Figure 2A,B).

### 2.3. Liraglutide Attenuated the LPS-Triggered Production of TNF-α and RANKL In Vivo

**Figure 3:**
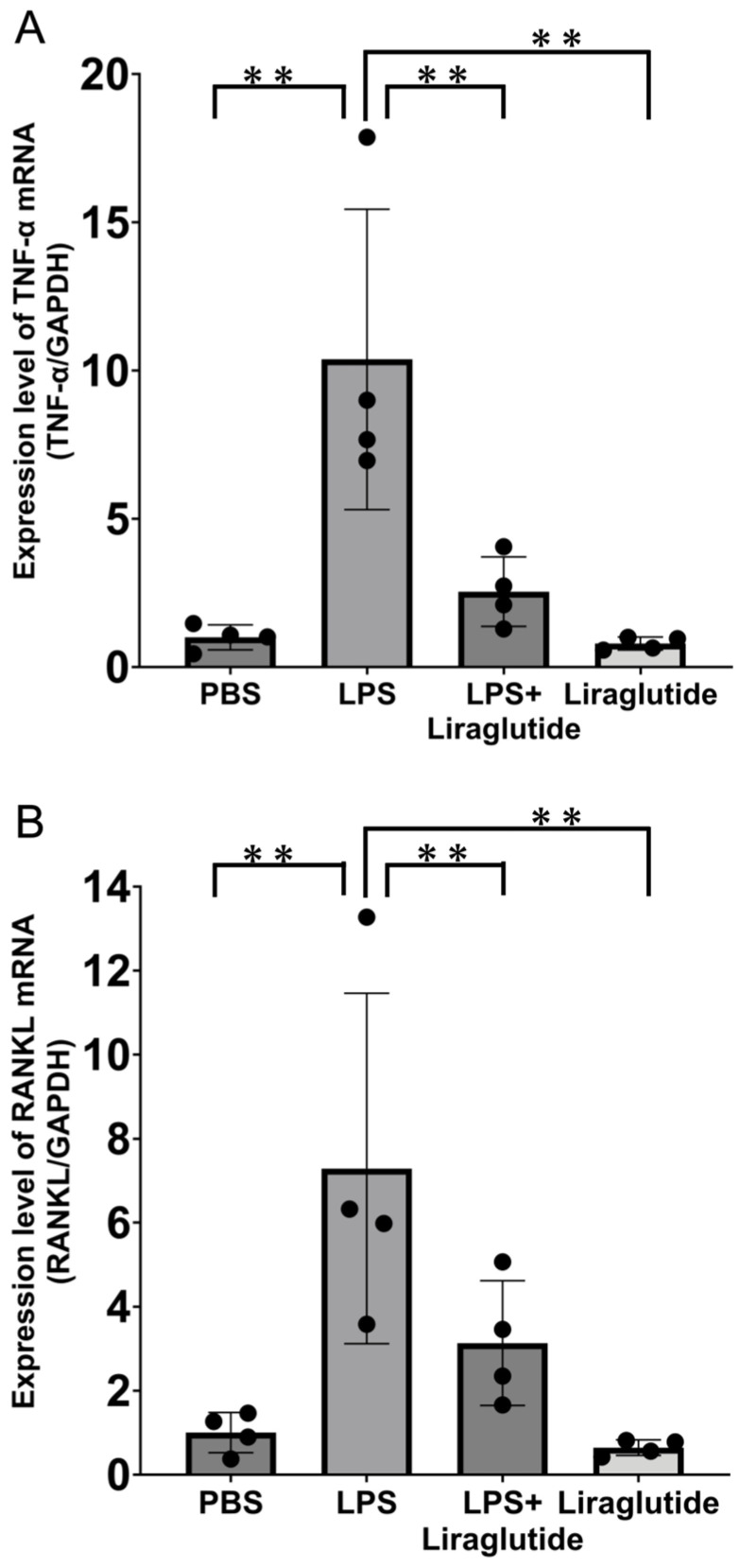
Liraglutide limited the LPS-induced mRNA upregulation of TNF-α and RANKL in vivo. Real-time RT-PCR was used to assess the expression profiles of key cytokines in mouse calvariae after five days of treatment with PBS, LPS, LPS + liraglutide, or liraglutide alone. (**A**) TNF-α and (**B**) RANKL mRNA levels were normalized against GAPDH. Statistics: one-way ANOVA followed by the Tukey–Kramer post hoc test; results are mean ± SD (n = 4; ∗∗ *p* < 0.01).

Quantitative real-time PCR analysis of murine calvarial bone demonstrated a robust inflammatory response following local LPS administration. This response was characterized by a marked induction of proinflammatory and osteoclastogenic transcripts, specifically TNF-α and RANKL, which were significantly upregulated in comparison to the vehicle-treated PBS. TNF-α mRNA expression increased from 1.00 ± 0.42 in the PBS group to 10.37 ± 5.07 in the LPS group and decreased to 2.54 ± 1.17 in the LPS + liraglutide group; RANKL mRNA expression increased from 1.00 ± 0.48 in the PBS group to 7.29 ± 4.17 in the LPS group and decreased to [3.13 ± 1.49] in the LPS + liraglutide group (Figure 3A,B). Notably, the concurrent administration of liraglutide served to attenuate this effect, effectively dampening the LPS-induced transcriptional surge of both markers TNF-α and RANKL (Figure 3A,B).

### 2.4. Liraglutide Did Not Influence RANKL- or TNF-α–Induced Osteoclast Formation, Precursor Cell Viability

**Figure 4:**
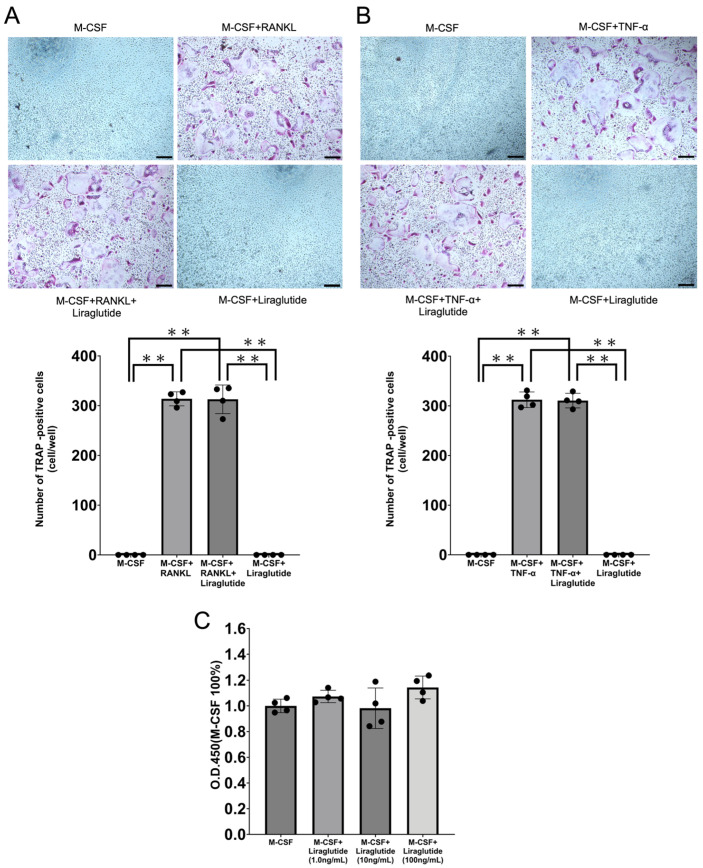
Liraglutide did not interfere with RANKL/TNF-α-driven osteoclastogenesis or precursor cell viability in vitro. Bone marrow-derived macrophages used as osteoclast precursors were obtained by culturing bone marrow cells with M-CSF for 72 h. (**A**,**B**) Microscopic images and corresponding cell counts of TRAP-positive cells following a 5-day incubation with M-CSF and either RANKL or TNF-α, with or without liraglutide. (**C**) Evaluation of osteoclast precursor viability after 5 d of exposure to M-CSF and varying doses of liraglutide (n = 4). Scale bar = 200 μm. Statistics: one-way ANOVA followed by the Tukey–Kramer post hoc test; results are mean ± SD (n = 4; ∗∗ *p* < 0.01).

To investigate whether liraglutide directly affects osteoclast precursor cells, we assessed its impact on RANKL- and TNF-α-induced osteoclast formation, as well as precursor viability. Both M-CSF/RANKL and M-CSF/TNF-α treatments yielded significant numbers of TRAP-positive cells. Notably, the addition of liraglutide did not alter these outcomes, as TRAP-positive cells remained prevalent in both experimental groups (313.75 ± 14.18 vs. 312.75 ± 28.83 cells/well). Similarly, no major changes in outcomes were observed between the M-CSF/TNF-α group and the M-CSF/TNF-α + liraglutide group (312.25 ± 15.65 vs. 310.5 ± 14.73 cells/well) (Figure 4A,B). Furthermore, cellular viability remained comparable across all groups, with no statistically meaningful variations detected after five days of culture; absorbance values of 1.00 ± 0.05, 1.07 ± 0.05, 0.98 ± 0.16, and 1.14 ± 0.09 were obtained for the control, 1, 10, and 100 ng/mL liraglutide groups, respectively (Figure 4C). Taken together, these data indicate that liraglutide exerts inhibitory effects through pathways other than the direct modulation of osteoclast precursor proliferation or differentiation. As shown in Figure 4C, the experiment was initially conducted to evaluate the cytotoxicity of liraglutide across various concentrations. Based on the finding that 100 ng/mL did not induce cytoxocity, this concentration was used for in vitro osteoclast formation assays.

### 2.5. Liraglutide Attenuated TNF-α Production in LPS-Stimulated Macrophages but Not LPS-Stimulated RANKL Expression in Osteoblasts

**Figure 5:**
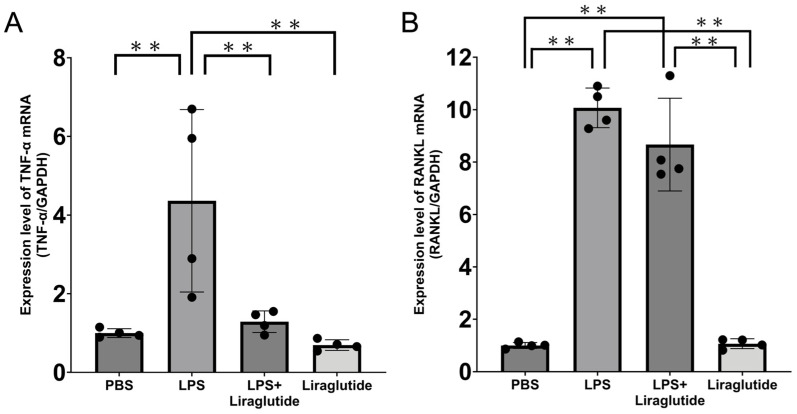
Liraglutide reduced TNF-α induced by LPS in macrophages but did not alter RANKL expression in osteoblasts in vitro. (**A**) TNF-α mRNA levels in peritoneal macrophages after 3 days of treatment with PBS, LPS, LPS + liraglutide, or liraglutide. (**B**) RANKL mRNA expression in osteoblasts under identical treatment conditions. Statistics: one-way ANOVA followed by the Tukey–Kramer post hoc test; results are mean ± SD (n = 4; ∗∗ *p* < 0.01).

Next, we examined whether liraglutide inhibits LPS-induced RANKL expression in osteoblasts in vitro. TNF-α mRNA levels in macrophages were analyzed using real-time RT-PCR. While LPS treatment significantly upregulated TNF-α expression in macrophages from 1.00 ± 0.11 in the PBS group to 4.36 ± 2.32, this effect was markedly attenuated by co-treatment with liraglutide, which reduced TNF-α expression 1.29 ± 0.27-fold relative to the PBS group (Figure 5A). To further investigate this mechanism, we assessed LPS-induced RANKL mRNA expression in osteoblasts. Although LPS significantly upregulated RANKL mRNA expression compared with the that in the control and liraglutide-only groups, increasing it from 1.00 ± 0.11 to 1.07 ± 0.19, the addition of liraglutide failed to attenuate this effect, and expression levels remained comparable to those in the LPS-only group (8.68 ± 1.79 vs. 10.10 ± 0.75) (Figure 5B). These results suggested that the inhibitory effect of liraglutide may not directly affect RANKL expression in osteoblasts.

### 2.6. Liraglutide Attenuated the LPS-Induced Activation of the MAPK Signaling Pathways in Peritoneal Macrophages

**Figure 6:**
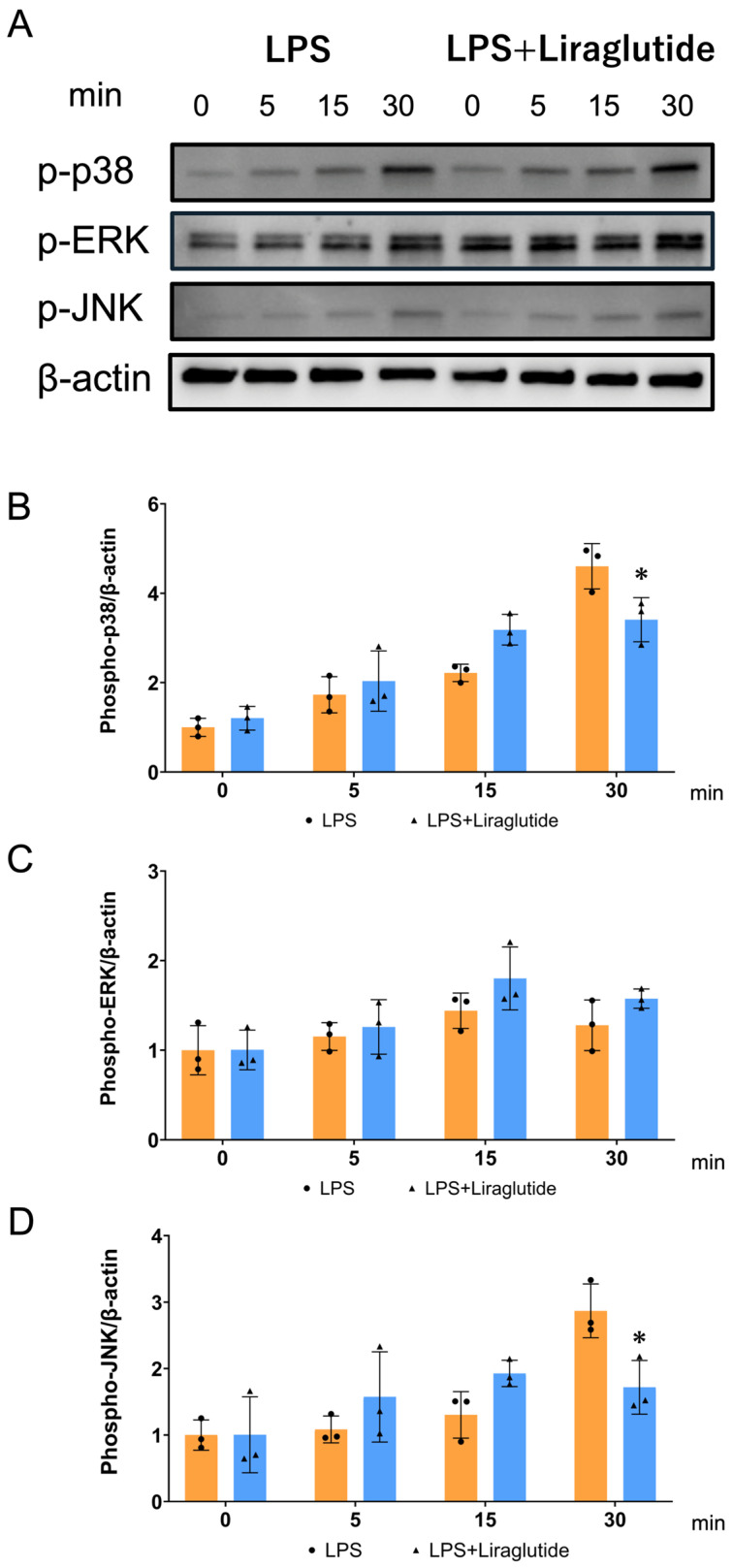
Liraglutide inhibited the LPS-induced activation of the MAPK signaling pathways in peritoneal macrophages. (**A**) Western blot analysis illustrating the impact of liraglutide on the phosphorylation of MAPK proteins relative to β-actin. (**B**–**D**) Densitometric quantification of the phosphorylated protein bands. Statistical significance was assessed using paired *t*-tests; non-significant results are not labeled (n = 3 for protein assays; ∗ *p* < 0.05).

To further clarify the molecular pathways by which liraglutide increases TNF-α expression in peritoneal macrophages, we investigated its impact on the MAPK signaling pathways, a critical regulator of the TNF-α. Peritoneal macrophages were treated with LPS, with or without liraglutide, and the activation levels of three MAPKs, JNK, p38, and ERK1/2, were analyzed using Western blotting (Figure 6A). The results revealed that liraglutide significantly attenuated the phosphorylation of JNK and p38 30 min after LPS stimulation, reducing their relative phosphorylation levels from 2.87 ± 0.40 to 1.72 ± 0.40 and from 4.60 ± 0.51 to 3.41 ± 0.49, respectively (Figure 6B,C). This suggests that liraglutide effectively inhibited the JNK and p38 pathways. However, liraglutide did not significantly alter ERK1/2 phosphorylation; the relative phosphorylation levels remained comparable between the LPS and LPS + liraglutide groups (1.33 ± 0.29 vs. 1.53 ± 0.28) (Figure 6D), indicating a selective rather than global inhibitory effect on MAPK signaling.

## 3. Discussion

This study aimed to elucidate the therapeutic potential of liraglutide in mitigating LPS-induced bone loss. In vivo assessments revealed that liraglutide treatment effectively inhibited the expansion of osteoclast populations and subsequent bone degradation. This improvement was closely associated with a marked reduction in the local expression of key proinflammatory mediators, specifically RANKL and TNF-α, which typically drive bone resorption. However, the mechanism underlying this protective effect appears to be regulatory rather than direct. When the study shifted to in vitro models, liraglutide failed to disrupt the differentiation of osteoclasts when they were directly stimulated by RANKL or TNF-α. Furthermore, liraglutide did not significantly affect the survival of osteoclast precursors nor did it alter the ability of osteoblasts to express RANKL in response to LPS. However, liraglutide was found to inhibited TNF-α expression induced by LPS in macrophages, suggested on indirect mechanism of action.

To maintain high metabolic stability and overcome rapid hormone degradation, a GLP-1 receptor agonist, exendin-4, was developed. Although endogenous GLP-1 is vital for glycemic control, it is neutralized within two minutes of secretion. To address this, exendin-4 was engineered with a specific amino acid sequence that remained stable against DPP-4-mediated degradation and functioned as a long-lasting GLP-1 receptor agonist for therapeutic use [39,41]. This resistance resulted in a significantly longer plasma half-life. Due to its prolonged half-life, optimized pharmacokinetics, and robust potency, exendin-4 is a promising candidate for clinical therapy [39,40]. Studies using GLP-1 receptor knockout models have demonstrated that this pathway is critical for suppressing osteoclast development and bone destruction [34]. In this study, we observed that the combined administration of LPS and liraglutide led to a marked decrease in bone loss compared to treatment with LPS alone. This demonstrates that liraglutide effectively suppresses LPS-triggered osteoclast development and resulting bone destruction in vivo. Here, liraglutide (20 μg/day) was administered via supracalvarial injection for five days. While previous rodent studies typically employed a dosage of 20 μg/kg daily over four weeks [41,54], we opted for a higher concentration to maximize the inhibitory effects. Throughout the experimental period, the mice were carefully monitored for any signs of systemic toxicity. No overt signs of systemic illness were observed in any of the treatment groups. Furthermore, no significant difference in body weight changes among the groups was observed, confirming that the administered doses of liraglutide and LPS did not cause significant morbidity (Supplementary Materials Figure S1). A previous report indicated that high doses of LPS can induce systemic toxicity and significant body weight loss [55]. While prolonged administration of these agents might lead to such adverse effects, our experimental timeline was limited to a short period of 5 days. This short-term model allowed us to successfully evaluate osteoclastogenesis induced by LPS without causing severe systemic morbidity. Within this brief timeframe, neither systemic illness nor significant cytotoxicity was observed. Additionally, liraglutide is well known for its pharmacological effect of inducing weight loss [55]. The short-term application of the high doses was considered appropriate and safe for the purposes of this study, but further investigations using clinically relevant doses are required.

Based on these results, we sought to elucidate the molecular pathways through which LPS-induced osteoclastogenesis and bone loss are suppressed. First, we explored whether liraglutide suppresses the LPS-induced expression of proinflammatory cytokines essential for osteoclast formation, specifically TNF-α and RANKL. Previous research has extensively documented that LPS triggers the production of both TNF-α and RANKL in vivo [42]. While RANKL remains the fundamental cytokine for osteoclastogenesis [56], research also identifies TNF-α as a potent inducer of osteoclastogenesis in vivo [17]. Consequently, a concurrent decrease in the levels of these cytokines is believed to inhibit osteoclastogenesis. In this study, TNF-α and RANKL mRNA upregulation triggered by LPS exposure was markedly diminished under concurrent liraglutide administration. These results imply that the reduction in LPS-driven osteoclastogenesis observed after liraglutide treatment was partially attributable to its ability to downregulate these essential inflammatory mediators. We further investigated whether liraglutide could directly inhibit RANKL- or TNF-α-stimulated osteoclastogenesis by acting on precursor cells. However, liraglutide failed to inhibit the differentiation of osteoclast precursors into mature osteoclasts, suggesting an indirect mechanism. Liraglutide has been shown to inhibit the differentiation of bone marrow macrophages and RAW264.7 cells into osteoclasts [52]. However, other studies failed to detect GLP-1 receptor expression in murine bone cells, such as osteoblasts and osteoclasts [53]. The absence of these receptors in osteoclasts makes it unclear whether liraglutide inhibits osteoclast formation via a direct cellular mechanism. In this study, liraglutide did not inhibit osteoclast formation in vitro, suggesting the lack of a direct cellular mechanism. Further investigation is required to reconcile these findings and clarify the inhibitory mechanism.

Furthermore, we investigated whether liraglutide affected the viability of osteoclast precursor cells. After five days of culture, no significant difference in cell viability was observed between the groups. These findings suggested that the inhibitory effect of liraglutide on osteoclastogenesis was not mediated by its direct effect on precursor cell survival. We further investigated whether liraglutide inhibits LPS-induced induction of RANKL expression in osteoblasts. However, liraglutide showed no direct inhibitory effects. We observed an inhibitory effect of liraglutide on TNF-α production in macrophages stimulated by LPS. Given that TNF-α drives osteoclastogenesis and stimulates RANKL production in osteoblasts, these results suggest that liraglutide’s ability to prevent in vivo osteoclast formation induced by LPS likely stems from the attenuation of macrophage-derived TNF-α and the subsequent downregulation of RANKL in osteoblasts. In a recent study, we explored the effects of liraglutide on osteoporosis, which is characterized by an inflammatory bone environment under oxidative stress. This causes macrophages to become M1 type, which release high level of TNF-α and induces destruct bone. Liraglutide acts by shifting these macrophages to the M2 type. Subsequently, TNF-α expression was reduced in macrophages [50]. Other study showed that liraglutide inhibits the polarization of macrophages into the M1 type induced by LPS and Interferon-gamma (IFN-γ) in rat experiment. By suppressing M1 polarization, liraglutide also significantly reduced the release of TNF-α in macrophages [57]. Our results are consistent with these findings. While previous studies failed to detect the GLP-1 receptor in murine osteoblasts and osteoclasts [53], we hypothesized that macrophage function may mediate the effects of liraglutide. To test this, we examined GLP-1 receptor expression in macrophages. The GLP-1 receptor mRNA expression was clearly detected in these cells (Supplementary Materials Figure S2), which strongly suggested that the beneficial effects of liraglutide on bone metabolism are not direct but are instead mediated indirectly through macrophage-driven mechanisms.

The MAPKs signaling cascade serves as a fundamental regulatory mechanism for an array of cellular processes, ranging from proliferation and differentiation to programmed survival. In inflammatory responses, this pathway is typically triggered by extracellular stressors [58]. Specifically, the activation and subsequent phosphorylation of its core components ERK1/2, p38, and JNK are critical drivers of the production and release of proinflammatory chemokines and cytokines in LPS-challenged macrophages [59]. To investigate whether liraglutide modulates these dynamics, we monitored the activation kinetics of the MAPK pathways in peritoneal macrophages. To eliminate any confounding variables associated with fetal bovine serum (FBS), the cells were first starved for a 3 h starvation period. Western blot analysis revealed that LPS induction triggered a sharp and rapid elevation in the phosphorylation levels of p38 and JNK proteins, peaking at the 30 min mark and ERK1/2 peaking at the 15 min mark. Notably, the introduction of liraglutide exerted a selective inhibitory effect; however, it significantly attenuated the LPS-induced phosphorylation of both JNK and p38 and did not appear to substantially alter the activation of ERK1/2. These findings suggest that the suppressive influence of liraglutide on JNK and p38 signaling may be the underlying mechanism responsible for the observed reduction in TNF-α expression within these macrophages. In macrophages, the synthesis of proinflammatory cytokines such as TNF-α is heavily dependent on the upstream activation and cascade phosphorylation of the JNK and p38 MAPK pathways [60]. For instance, the specific pharmacological inhibition of JNK and p38 phosphorylation has directly induced a significant decrease in TNF-α expression in macrophage models [61]. In agreement with earlier reports, the marked suppression of both JNK and p38 MAPK phosphorylation observed in our study subsequently led to a reduction in downstream TNF-α expression.

Our findings demonstrated that liraglutide effectively suppressed LPS-induced bone resorption and osteoclast formation triggered by LPS. These observations indicate that liraglutide has significant potential as a therapeutic agent for preventing inflammation-driven skeletal degradation. Although the data are promising, the reliance on a localized injection protocol means that the study does not account for the complex systemic distribution and pharmacokinetics typically observed in humans. Therefore, discretion is required when applying specific animal model outcomes to systemic human treatments. Despite this, the biological mechanisms identified in this study offer a compelling rationale for investigating how systemic GLP-1 receptor agonists protect bone integrity in clinical settings. Moreover, our experiments were performed on healthy subjects, and subsequent research must determine whether these bone-protective effects persist under pathological states such as diabetes, where incretin-based therapies are standard. As the first study to directly analyze the impact of exogenous liraglutide in this specific inflammatory context, this study offers unique perspectives on GLP-1 signaling. Transitioning from local models to systemic investigations is critical for fully elucidating the role of the GLP-1 pathway in maintaining bone health during inflammatory challenges. Furthermore, given that liraglutide is a therapeutic for type 2 diabetes, which is characterized by impaired bone quality, hyperglycemia, and chronic inflammation, future studies using murine diabetes models are required to confirm its clinical relevance.

## 4. Materials and Methods

### 4.1. Animals and Reagents

Male C57BL6/J mice (8–10 weeks old) were obtained from CLEA Japan (Tokyo, Japan) and housed at our animal facility. All experimental protocols, including handling, treatment, and maintenance, were conducted under the rigorous ethical standards and institutional guidelines mandated by Tohoku University (Approval No. 2018DnA-049-13). Every effort was made to minimize animal discomfort and ensure humane treatment. *Escherichia coli* LPS and liraglutide were purchased from Sigma-Aldrich (St. Louis, MO, USA). Animals were randomly assigned to experimental groups (n = 4 per group).

### 4.2. Histological Analysis

To investigate osteoclastogenesis, we utilized a murine calvarial model following a previously reported method [42]. The experimental subjects were divided into four cohorts: a negative control group receiving PBS, positive control group administered 100 μg of LPS daily via subcutaneous injection at supracalvaria, treatment group receiving both LPS and liraglutide (20 μg/day), and final group treated solely with liraglutide. The induction protocol involved five consecutive days of injection, after which the mice were euthanized on the sixth day for tissue collection. Upon excision, the calvariae were preserved in 4% PBS-buffered formaldehyde at 4 °C overnight and then subjected to a three-day demineralization process in 14% ethylenediaminetetraacetic acid (EDTA) at room temperature with gentle shaking, during which the EDTA solution was refreshed daily. For histological evaluation, the specimens were partitioned into three segments perpendicular to the sagittal suture, embedded in paraffin wax, and sliced into 5 μm sections. To identify the osteoclasts, we performed TRAP staining and hematoxylin counterstaining. The following quantitative criteria [62]. TRAP-positive cells with a minimum of three nuclei within the mesenchyme of the sagittal suture were counted to determine the extent of osteoclast formation across groups.

### 4.3. Osteoclast Precursor Preparation for Osteoclast Differentiation

Primary bone marrow cells were harvested from the femurs and tibias of C57BL/6J mice using strict aseptic techniques. The marrow was liberated by flushing the medullary cavities with α-MEM (Sigma-Aldrich) using a 25-gauge needle, after which the resulting cell suspension was passed through a 40 μm nylon filter and concentrated via centrifugation at 3000× *g* for 3 min. These cells were initially maintained in α-MEM supplemented with 10% FBS, antibiotics (penicillin/streptomycin; FUJIFILM Wako Pure Chemical Corporation, Osaka, Japan), and M-CSF in a humidified atmosphere with 5% CO_2_ at 37 °C. To enrich osteoclast progenitors, the culture was depleted of non-adherent cells by washing with PBS, and the remaining adherent cells were detached using trypsin-EDTA (Gibco, Grand Island, NY, USA). The isolated precursors were then seeded and maintained in M-CSF-supplemented medium for subsequent experimental use [63]. To investigate the regulatory effects of liraglutide on osteoclast formation, these precursors were seeded into 96-well plates at a density of 5 × 10^4^ cells per 200 μL of medium. The cells were cultured for five days under various conditions: a baseline group with M-CSF (100 ng/mL) alone, and stimulatory groups receiving M-CSF in conjunction with either RANKL (Gibco) or TNF-α (R&D Systems, Minneapolis, MN, USA) (100 ng/mL each). In parallel, the effect of liraglutide (100 ng/mL) was assessed by adding it to M-CSF medium in the presence or absence of the two osteoclastogenic cytokines. The culture medium was refreshed every two days. Following incubation, the cells were fixed in 10% formalin for 30 min and permeabilized using 0.2% Triton X-100 (Sigma-Aldrich) for 5 min. Osteoclast differentiation was identified by TRAP staining and mature osteoclasts, characterized by TRAP-stained cells containing at least three distinct nuclei, were quantified using light microscopy.

### 4.4. Osteoblast Preparation

To isolate primary osteoblasts, calvariae were dissected from 5- to 6-day-old mice and subjected to sequential digestion. An isolation buffer was prepared containing 60 mM sorbitol, 70 mM NaCl, 3 mM K_2_HPO_4_, 10 mM NaHCO_3_, 1 mM CaCl_2_, 0.1% (*w*/*v*) bovine serum albumin (BSA; Sigma-Aldrich), 0.5% (*w*/*v*) glucose, and 25 mM HEPES. The digestion utilized a 0.2% (*w*/*v*) collagenase solution formulated in this buffer, alongside a 5 mM EDTA solution containing 0.1% BSA in PBS, which was sterilized using a 0.2 μm filter. Calvariae were processed through five successive digestion steps at 37 °C on a shaker at 300 rpm: collagenase for 20 min (fraction 1), EDTA for 15 min (fraction 2), collagenase for 20 min (fraction 3), collagenase for 20 min (fraction 4), and EDTA for 15 min (fraction 5). While all the digests were collected, fractions 3–5 contained the highest concentrations of osteoblasts. During the sequential enzymatic digestion process, cells were released from the bone matrix in a phase-dependent manner. All digests were collected and analyzed; fractions 3–5 contained the highest concentrations of osteoblasts. This distribution was attributed to the initial fractions (1–2) predominantly releasing surrounding periosteal cells and fibroblasts, while the subsequent fractions (3–5) successfully digested the deeper, mineralized matrix, thereby isolating a highly enriched population of mature, functional osteoblasts. Following collection, the cells were cultured overnight in growth medium. Adherent cells were harvested using trypsin-EDTA, seeded at a density of 2 × 10^5^ cells/cm^2^, and cultivated for an additional three days; the medium was replenished every two days. The resulting adherent cell population served as primary osteoblasts for subsequent experiments [63].

### 4.5. Peritoneal Macrophage Extraction

Resident peritoneal macrophages were isolated from the mice under resting conditions. Briefly, the peritoneal cavity was injected with 5 mL of sterile ice-cold PBS using a 21-gauge needle. The abdomen was gently massaged for 1 min to suspend the cells, and the fluid was aspirated to collect the peritoneal cells. These cells were centrifuged at 1500× *g* for 8 min at 4 °C and washed twice with α-MEM (Sigma-Aldrich) supplemented with 10% FBS. After an initial 1 h incubation period, non-adherent cells were removed. After 24 h of culture, the remaining adherent cells were harvested and used as macrophages in subsequent experiments, following a well-established standard protocol for macrophage enrichment [64].

### 4.6. RNA Extraction and Expression Analysis

Following the protocols used for the in vivo experiments, calvarial tissues were collected, flash-frozen in liquid nitrogen, and homogenized with 800 μL of TRIzol reagent (Invitrogen, Carlsbad, CA, USA) using a Tomy Seiko Micro Smash MS-100R (Tomy Seiko, Tokyo, Japan). Total RNA was purified from the cell lysates using an RNeasy Mini Kit (Qiagen, Valencia, CA, USA). For the in vitro assessments, osteoblasts and macrophages were maintained in growth media containing PBS, 100 ng/mL LPS, a combination of LPS and 100 ng/mL liraglutide, or liraglutide alone. Following a 72 h incubation period, the RNeasy Mini Kit was employed to extract total RNA from the attached cell cultures. First-strand cDNA was subsequently generated from 2 μg of this isolated RNA template in a final volume of 20 μL, incorporating reverse transcriptase and oligo-dT primers. The target mRNA expression was then examined via real-time RT-PCR utilizing a Takara Thermal Cycler Dice Real-Time system. For each qPCR assay, a 25 μL mixture was prepared by combining 2 μL of the synthesized cDNA with 23 μL of a master mix consisting of SYBR Premix Ex Taq (Takara, Shiga, Japan) and 50 pmol/μL of the appropriate gene-specific primers. For the amplification protocol, initial denaturation was carried out at 95 °C for 10 s, followed by 45 to 60 subsequent cycles. Each cycle consisted of a 5 s denaturation step at 95 °C and a combined annealing and extension step at 60 °C for 30 s. The relative abundance of each mRNA species was determined by comparison with GAPDH expression with previously reported primer sequences [63].

### 4.7. Micro-CT Quantification of Bone Destruction

Immediately after euthanasia, the calvariae of the mice were surgically excised and subjected to stabilization. This involved immersing the harvested bone tissues in a 4% solution of formaldehyde buffered with PBS for a duration of 72 h, maintained at a consistent temperature of 4 °C. Once the fixation period was completed, a rigorous rinsing protocol using PBS was implemented to ensure the removal of any residual fixative. To facilitate a detailed assessment of the bone resorption pits, the specimens were analyzed by high-resolution microfocus computed tomography using the ScanXmate-E090 system (Comscan, Kanagawa, Japan). The raw data obtained from these scans were then processed to generate sophisticated three-dimensional volumetric models using TRI/3D-BON64 specialized software (RATOC System Engineering, Tokyo, Japan). The final stage of the morphometric analysis involved the quantitative evaluation of the resorptive activity. Using ImageJ software (version 1.54g; National Institutes of Health, Bethesda, MD, USA), the precise surface area of the resorption pits was measured and expressed as a proportional percentage of the total surface area. This metric serves as the primary indicator for characterizing the extent of osteoclastic bone degradation across samples [63].

### 4.8. Osteoclast Precursor Survival and Proliferation Assessment

To assess cellular metabolic activity and survival, osteoclast precursors were inoculated into 96-well microplates, achieving an initial seeding density of 10,000 cells per well within a total volume of 200 μL of medium. To initiate the experiment, the culture was supplemented with 100 ng/mL M-CSF. This was conducted across two distinct groups: one receiving additional treatment with 1, 10, or 100 ng/mL liraglutide, and a control group maintained without liraglutide. After maintaining the cultures in a controlled environment for five days, the spent media were discarded. To remove any residual treatment factors or metabolic waste, cells were washed thoroughly with PBS. Subsequently, the wells were replenished with 100 μL of pristine culture medium. Each variable was tested using four independent replicates to ensure statistical reliability and minimize experimental error. The quantification of viable cells was performed by introducing 10 μL of Cell Counting Kit-8 (CCK-8) reagent (Dojindo Molecular Technologies, Kumamoto, Japan) into each individual well. Following a final 2 h incubation period at 37 °C, the optical density was measured. The resulting absorbance values were measured at 450 nm using a specialized microplate spectrophotometer, where the intensity of the signal served as a direct proxy for the proportion of living cells in each treatment group after subtracting the background absorbance of cell-free blanks [63].

### 4.9. Western Blot

To assess the signaling response, peritoneal macrophages were first subjected to 3 h of starvation in serum-free α-MEM before being stimulated with LPS, with or without the addition of liraglutide, across a time course of 0, 5, 15, and 30 min. After stimulation, the cells were harvested in RIPA buffer (EMD Millipore Corp., Billerica, MA, USA) containing 1% protease and phosphatase inhibitors (Thermo Fisher Scientific, Waltham, MA, USA), and the resulting lysates were processed through a QIAShredder (Qiagen) at 14,000 rpm for 2 min at 4 °C to achieve homogenization and eliminate debris. Protein concentrations were determined by measuring absorbance at 595 nm using a BCA assay kit (Thermo Fisher Scientific, Waltham, MA, USA) and a microplate reader, after which the samples were either stored at −80 °C or prepared for analysis by mixing with a loading buffer consisting of 4× Laemmli (Bio-Rad Laboratories, Hercules, CA, USA) and 2-mercaptoethanol in a 9:1 ratio at a 3:1 sample-to-buffer volume ratio. These mixtures were denatured at 95 °C for 5 min and maintained at −20 °C until electrophoresis was performed using Bio-Rad Precast Gels (Bio-Rad Laboratories) in 1× Tris/Glycine/SDS buffer at 120V for 1 h. After separation, the proteins were transferred onto PVDF membranes using the Trans-Blot Turbo Transfer System (Bio-Rad Laboratories) and blocked using TBS-T solution (1% Triton X-100) containing 4 g Block Ace (KAC Co., Ltd., Kyoto, Japan) and 20 mg sodium azide for 1 h at room temperature. The membranes were then incubated overnight at 4 °C with primary antibodies against Phospho-p44/42 (Erk1/2), Phospho-p38, and Phospho-SAPK/JNK (all from Cell Signaling Technology, Danvers, MA, USA), alongside an anti-β-actin (Sigma-Aldrich) internal control. Following three washes in TBS-T for 10 min each, a secondary incubation was performed for 1 h at room temperature using horseradish peroxidase-conjugated anti-rabbit or anti-mouse antibodies (Cytiva, Marlborough, MA, USA). Finally, the protein bands were developed with the West Femto Maximum Sensitivity Substrate (Thermo Fisher Scientific), imaged using the FUSION FX and Evolution-capt Edge system (VILBER, Collégien, France), and quantified using ImageJ software employing an integrated band quantification macro [63].

### 4.10. Statistical Analysis

Data are presented as means ± standard deviation (SD). Statistical significance between groups was evaluated using the Tukey–Kramer post hoc test and paired *t*-tests, with a *p*-value of less than 0.05 considered statistically significant.

## 5. Conclusions

**Figure 7:**
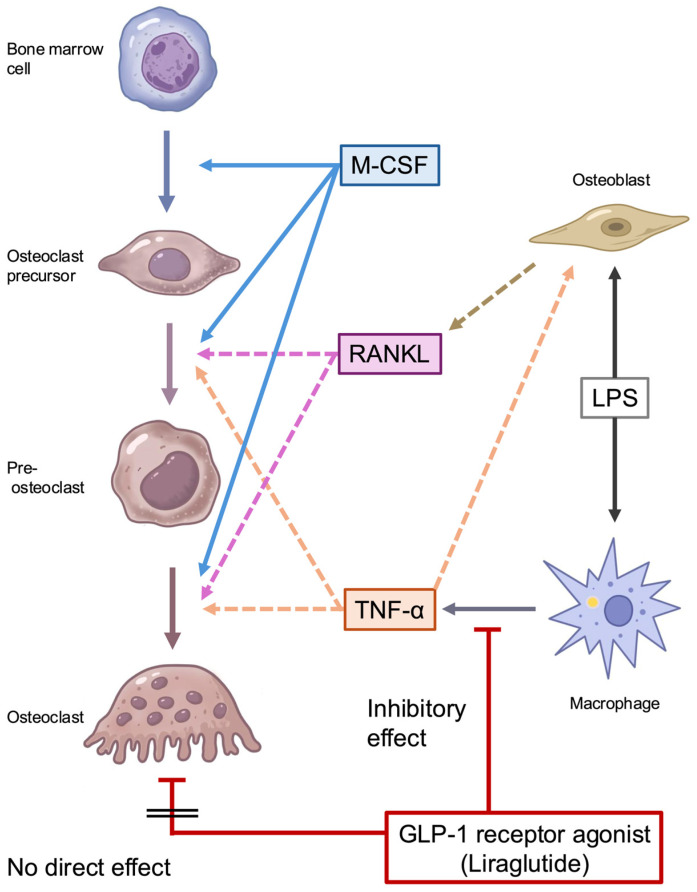
Proposed mechanism through which Liraglutide suppresses LPS-induced osteoclastogenesis in vivo by reducing macrophage-derived TNF-α. Because LPS triggers the release of RANKL from osteoblasts and TNF-α from peritoneal macrophages, both of which are critical drivers of osteoclast formation, liraglutide effectively disrupts the differentiation of precursors into mature osteoclasts. Although liraglutide did not directly interfere with RANKL- or TNF-α-mediated osteoclast formation, it significantly inhibits LPS-induced TNF-α production in macrophages. Given that TNF-α both drives osteoclastogenesis and triggers further RANKL production in osteoblasts, these findings suggest that liraglutide prevents bone loss in vivo by suppressing macrophage-derived TNF-α, thereby downregulating the subsequent RANKL signaling cascade.

The protective effect of liraglutide against bone loss appears to be mediated through an indirect systemic pathway rather than through the direct inhibition of osteoclast differentiation. Under inflammatory conditions triggered by LPS, the region undergoes a dual-pronged activation of bone-resorbing factors: LPS induces the release of RANKL from osteoblasts while simultaneously stimulating macrophages to secrete TNF-α. These two cytokines act as primary catalysts driving the transition of osteoclast precursors to bone-resorbing osteoclasts. While liraglutide does not possess the capacity to halt osteoclast formation when RANKL or TNF-α are already present, its therapeutic value lies in its ability to intervene at the source of inflammation. Specifically, liraglutide exerts a suppressive effect on macrophage-derived TNF-α production following an LPS challenge. This intervention is critical because TNF-α functions as a potent upstream regulator; it both promotes osteoclastogenesis and amplifies the inflammatory cycle for osteoblasts to produce additional RANKL. These findings suggest a hierarchical mechanism by which liraglutide acts as an immunomodulatory agent. By dampening the initial macrophage response and curtailing the subsequent TNF-α induced RANKL, liraglutide effectively mitigates the pathological bone resorption typically observed in vivo during inflammatory states (Figure 7).

## Data Availability

The original contributions of this study are included in this article. Further inquiries can be directed to the corresponding author.

## Supplementary Materials

The following supporting information can be downloaded at https://www.mdpi.com/article/10.3390/ijms27125624/s1.
