# Supplementary material for: Liraglutide, a GLP-1 Receptor Agonist, Mitigates LPS-Induced Osteoclastogenesis and Bone Loss by Downregulating Macrophage TNF-α Expression
Source: Int J Mol Sci. 2026 Jun 22;27(12):5624. doi: 10.3390/ijms27125624 (PMC13299069; doi:10.3390/ijms27125624)
Supplement: Supplementary file 1 [file ijms-27-05624-s001.zip › Supplementary Figure.pdf]

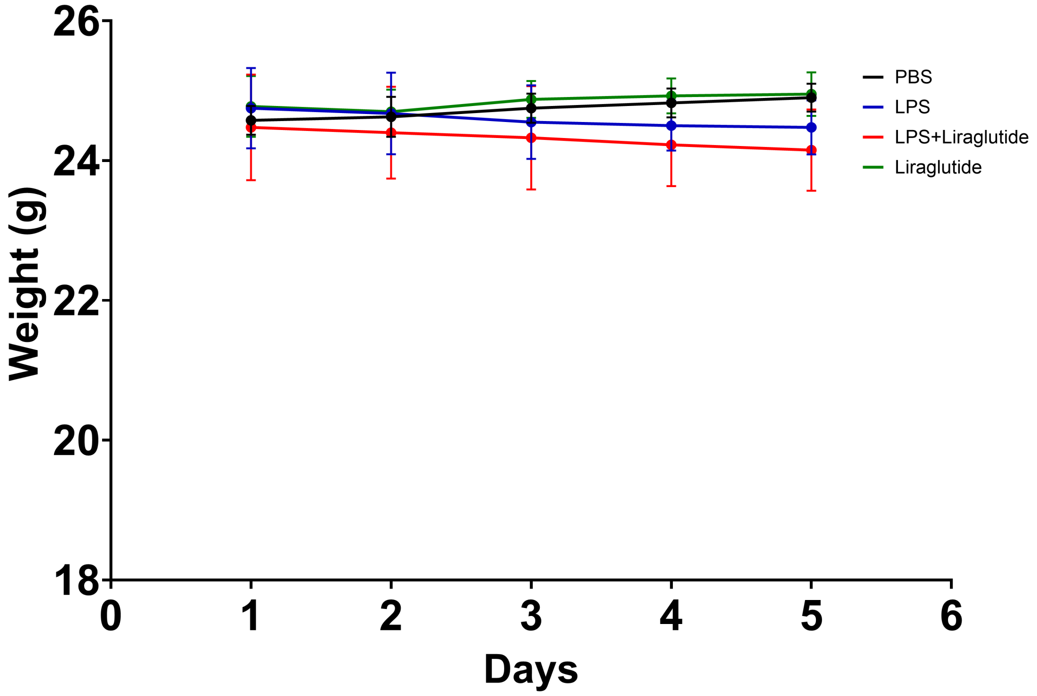


Supplementary Materials Figure S1. Daily body weight changes following LPS and liraglutide administration.

Mice were randomly divided into four groups and treated daily for 5 days as follows: a negative control group receiving PBS; a positive control group administered 100 µg/day of LPS via subcutaneous injection at the supracalvaria; a treatment group receiving both LPS (100 µg/day) and liraglutide (20 µg/day); and a final group treated solely with liraglutide (20 µg/day). Body weights of the mice were measured daily. Evaluation of daily body weight changes revealed no significant differences among the four groups. While the PBS and liraglutide-alone groups showed a gradual increase in body weight, almost no weight gain was observed in the LPS and LPS+liraglutide co-administration groups.

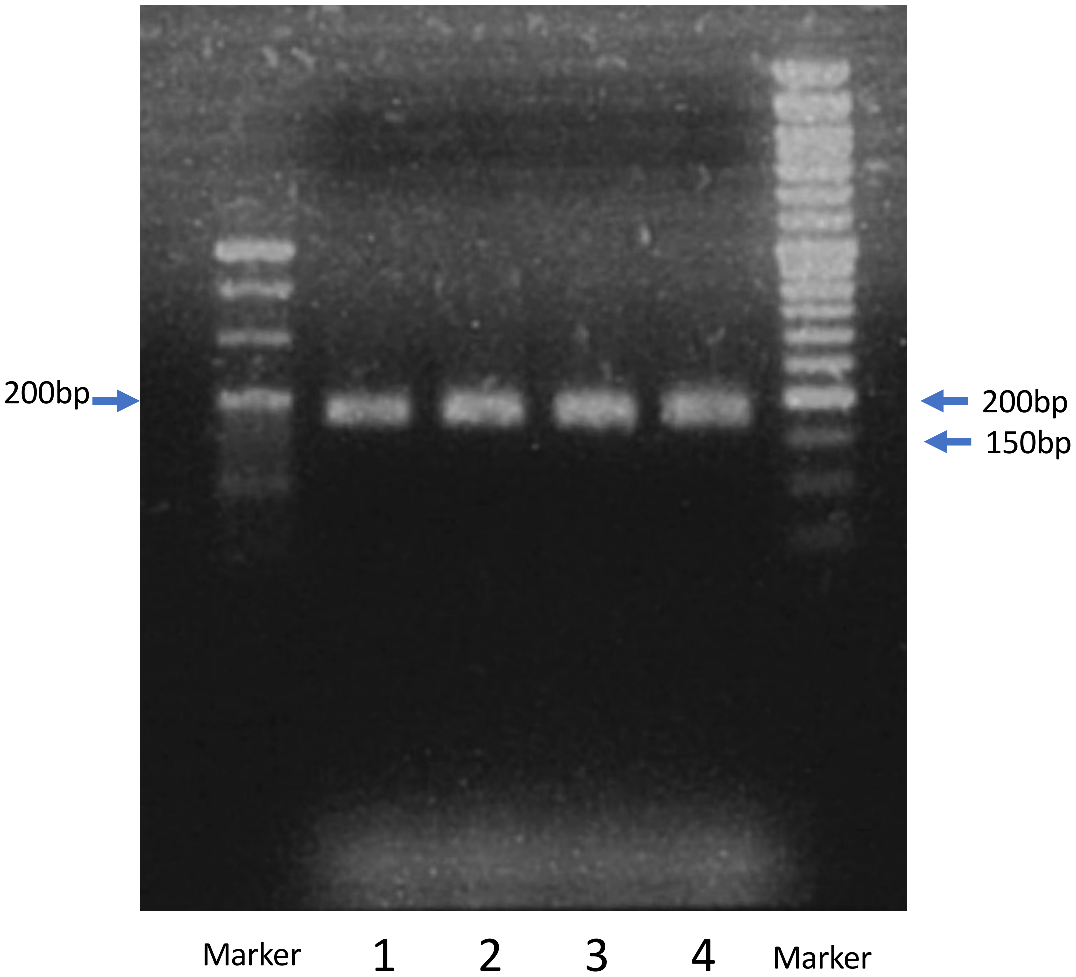


Supplementary Materials Figure S2. Expression of GLP-1 receptor in peritoneal macrophages.

Resident peritoneal macrophages were isolated from 4 mice and processed individually. Total RNA was isolated from each peritoneal macrophage. For RT-PCR analysis, cDNAs were synthesized from 1 μg of total RNA. RT-PCR was performed in a 50 μl reaction volume containing 2 μl of cDNA and specific primers (50 pmol each) using Ex Taq (Takara Bio Inc., Shiga, Japan) according to the manufacturer's instructions. The primers used were as follows: 5′-GGGTCTCTGGCTACATAAGGACAAC-3′ and 5′-AAGGATGGCTGAAGCGATGAC-3′, which yielded a 178-bp amplicon. Samples were preheated to 94°C and subjected to 35 PCR cycles for GLP-1 receptor. Each cycle consisted of a denaturation step at 94°C for 30 s, an annealing step at 55°C for 30 s, and an extension step at 72°C for 1 min. Aliquots (10 μl) of the PCR products were separated by electrophoresis in a 2.0% agarose gel. Electrophoresis confirmed the amplification of the 178-bp fragment from each of the four samples.
